# Household income and obesity among older adults: the moderating role of race in a longitudinal analysis

**DOI:** 10.1186/s12889-025-22910-1

**Published:** 2025-10-02

**Authors:** Sunkanmi Folorunsho, Victor Ajayi, Munirat Sanmori

**Affiliations:** 1 Department of Sociology, University of Nebraska-Lincoln, Lincoln, NE USA; 2 School of Social Work, University of North Carolina at Chapel Hill, Chapel Hill, USA; 3 Department of Sociology, Georgia State University, Georgia, Atlanta USA

**Keywords:** Obesity, Older adults, Household income, Race/Ethnicity, Health

## Abstract

**Background:**

Obesity among older adults in the United States is a growing public health concern, with rising rates contributing to chronic disease, disability, and premature mortality. While higher income is generally associated with lower obesity risk, this relationship may not hold equally across racial and ethnic groups. This study examines how household income relates to obesity among older adults and whether race moderates this association.

**Methods:**

We used longitudinal data from the Health and Retirement Study (2014–2018), a nationally representative panel of U.S. adults aged 50 and older (*N* = 12,118). Obesity was defined as BMI ≥ 30 using self-reported height and weight. Household income was measured both continuously and in quartiles. We estimated mixed-effects logistic regression models with race-income interaction terms, adjusting for age, gender, nativity, work status, functional limitations, and survey year.

**Results:**

Higher income was associated with reduced odds of obesity overall (OR = 0.95 per $10,000, 95% CI: 0.92–0.99), but this protective effect differed by race. Among White older adults, income was strongly protective; among Black adults, income had no significant effect (interaction OR = 1.05, *p* < 0.05); and among Hispanic adults, the effect was weaker and non-significant. Obesity disparities persisted across income levels, with high-income Black seniors having higher obesity prevalence than low-income Whites.

**Conclusions:**

Income-based health advantages do not accrue equally across racial groups. Interventions must go beyond economic measures to address structural barriers, cultural contexts, and life-course disadvantage that shape obesity risk in later life.

## Introduction

Obesity in the older adult population has become a significant public health concern in the United States. In recent decades, obesity rates among older Americans have risen sharply, nearly doubling from 22% in the early 1990s to about 40% by 2015–2018 [1]. This means that nearly two out of every five Americans aged 65 and older are now classified as obese, signaling a notable generational shift in body weight patterns. This trend is particularly concerning because persistent obesity in later life is associated with heightened risks of chronic illnesses such as diabetes and cardiovascular disease, as well as increased rates of disability, premature mortality, higher healthcare costs, and a greater likelihood of requiring nursing home care [1].

A robust body of literature indicates that obesity is not evenly distributed across society. Instead, it is shaped by social disparities [2]. In the U.S, older adults from racial and ethnic minority groups and those with lower socioeconomic status (SES) bear a disproportionate burden of obesity and related health problems [1, 3]. For instance, national data show that obesity prevalence is higher among Black and Hispanic adults than among non-Hispanic White adults in mid- to late-life [1]. These disparities reflect the cumulative impact of social and economic disadvantages that often begin early in life and persist into older age [4]. Black and Hispanic populations have historically faced challenges such as lower access to quality education and high-paying jobs, higher rates of poverty, experiences of discrimination, and residence in neighborhoods with fewer health-promoting resources [5, 6]. Such social disparities can create lifelong differences in health behaviors and outcomes. We define social disparities as the inequitable social conditions and structural factors (for instance, poverty, discrimination, residential segregation) that lead to worse health outcomes for certain groups [6, 7]. In the context of obesity, social disparities might manifest as limited access to healthy foods or safe exercise environments in low-income or predominantly minority communities, chronic stress from economic hardship or racism that disrupts metabolism, and less access to healthcare for weight management [8].

Household income is one of the most important socioeconomic indicators related to health in later life. Higher income can facilitate better nutrition, safer housing, access to fitness opportunities, and quality healthcare, which should, in theory, lead to better health outcomes including healthier body weight [6]. At younger ages, an inverse relationship between SES and obesity is often observed, especially among women: those with higher income or education tend to have lower obesity risk [9]. However, this relationship can be complex and may differ by race and gender [9].

Prior research has found that the protective effect of high SES on obesity is not uniform across racial groups. For example, among White women, higher income or education is strongly associated with lower obesity prevalence, but among Black women this gradient is much weaker or even absent [10]. In one study, Black women with a college education had obesity rates that were as high as those of less-educated Black women, whereas White women showed sharp declines in obesity with greater education [10]. Similar patterns have been observed with income: while higher family income is generally associated with lower odds of obesity among White individuals, studies have found little to no reduction in obesity risk with increased income among Black individuals [9, 11]. These disparities suggest that race and ethnicity may moderate the relationship between income and obesity. In other words, the health benefits typically linked to higher socioeconomic status may not be equally experienced across racial and ethnic groups [1, 12].

Older adults present a unique context to examine these issues. First, late life is the culmination of decades of exposures and behaviors, so an individual’s health status (including body weight) at older ages reflects life-course processes [13]. Theories of aging like cumulative disadvantage or cumulative inequality theory explain that social and health inequalities tend to build up over time, resulting in significant gaps in well-being during old age [14]. Rather than diminishing with age, these disparities often grow larger, as early-life advantages or disadvantages become more pronounced over the course of a person’s life [15]. In line with Geronimus’s weathering hypothesis, Black Americans experience accelerated health decline due to chronic social adversity, resulting in earlier onset of health problems and premature aging of physiological systems [16]. By the time people reach older adulthood, those from long-disadvantaged groups (e.g. Black and Hispanic individuals who faced lifelong discrimination and economic hardship) may exhibit higher rates of obesity, diabetes, hypertension, and other conditions [17]. These disparities are not simply explained by current income or health behaviors, because the wear and tear of cumulative stress (“weathering”) and structural barriers may limit the returns of socioeconomic gains in adulthood [18]. Second, older adults have distinct health vulnerabilities: obesity in this age group can contribute to functional impairments, such as difficulty with mobility and daily activities, and can worsen age-related conditions like arthritis. Research shows that older adults who are obese are significantly more likely to develop new disabilities (difficulty in Activities of Daily Living) over time compared to their normal-weight peers [3].

### Theoretical frameworks and application to older adults

Our study is informed by two primary theoretical frameworks: (1) the economic health capital model proposed by Michael Grossman, and (2) the social determinants of health paradigm. Grossman’s model conceptualizes health as a form of capital that individuals build and depreciate over time. People invest in their health (through medical care, nutrition, exercise, etc.) and these investments are constrained by resources like income and education [18]. A key insight from Grossman is that health decisions have a lifetime horizon– investments (or lack thereof) early in life can have lasting impacts in later years. Because the model explicitly considers a lifetime perspective, it is well-suited to understanding health outcomes in an aging population [18]. In this framework, higher income allows more health investment at all ages, which should lead to better health and lower likelihood of obesity by old age [19]. Indeed, if a 70-year-old individual has consistently had high income, the model would predict they likely had better access to healthy food and healthcare throughout adulthood, leading to lower cumulative weight gain. Grossman’s theory would therefore anticipate an inverse income-obesity relationship even among older adults, as long as those income differences have persisted over time. That said, the model primarily focuses on individual agency and resource allocation; it assumes that individuals maximize their health given their constraints [20]. This may be an oversimplification for older adults of different races if we consider the broader context. For example, a high-income Black senior may have faced barriers or stressors that a high-income White senior did not, meaning that equal financial resources did not yield equal ability to invest in health. This is where the social determinants of health framework augments our understanding.

The social determinants of health (SDoH) framework, as defined by the World Health Organization and adopted by public health research, emphasizes that “the conditions in which people are born, grow, live, work, and age” are fundamental drivers of health inequalities [21]. These conditions include factors like socioeconomic status, education quality, neighborhood environment, healthcare access, social support, and discrimination. Rather than health being solely a product of personal choices and medical care, SDoH points to how contextual factors shape opportunities and exposures. For obesity, this means looking at food environments, social norms, stress, and recreation opportunities as mentioned above. In applying SDoH to older adults, we note that today’s older population has lived through decades of social change and differing social policies [22]. Many of those who are old now (for instance, those in their 70s and 80s) may have grown up in eras of legal segregation or limited civil rights, which could have long-lasting effects on their socioeconomic trajectory and health. Furthermore, aging itself can change individual’s social determinants: retirement may reduce income and social contact; losing the ability to drive can alter one’s access to grocery stores or community events [23]. Thus, when we consider older adults, we must consider not just current social conditions but the accumulated influence of social conditions over a life course.

In the context of our research question, these frameworks led us to specific expectations: We anticipated that overall, higher income will be associated with lower odds of obesity in older adults (consistent with Grossman’s model and general SES-health gradients). However, drawing on SDoH and life-course perspectives, we also expected that this association will be moderated by race. Specifically, due to structural and systemic factors, the income-health gradient may be attenuated among Black and Hispanic older adults. Higher income might not confer the same magnitude of protective effect for minorities if, for instance, even affluent Black and Hispanic individuals face unique stressors or if health-promoting resources are not as accessible in the communities where many minority seniors reside. Our study focused on race-by-income interactions, controlling for gender, to isolate how the income effect differs by racial/ethnic group in older age.

## Methods

### Data and sample

We used data from the Health and Retirement Study (HRS), a longitudinal panel study of U.S. adults over age 50. The HRS, which began in 1992, surveys a nationally representative cohort of older Americans every two years [24]. The study employs a multistage area probability sample with oversampling of Black and Hispanic individuals, ensuring sufficient representation of these groups. HRS collects extensive information on respondents’ health status, economic circumstances, employment, and family dynamics [24], making it well-suited for our analysis which spanned both health (obesity) and socioeconomic (income) domains. For this study, we focused on the three recent public survey waves with available data on our key variables: 2014, 2016, and 2018. We selected this period to capture contemporary trends in obesity among older adults and to follow individuals across multiple time points in the mid-2010s.

### Measures

#### Outcome variable

Our outcome of interest was obesity status, assessed at each wave. Obesity is defined as body mass index (BMI) ≥ 30 kg/m², based on self-reported height and weight [25]. In each interview, respondents were asked to report their current weight and their height. BMI was calculated as weight in kilograms divided by height in meters squared. We recognized that self-reported weight can underestimate true weight, especially among heavier individuals; however, validation studies in older adults have found self-reports reasonably correlate with measured values, with slight underestimation on average [26]. We did not have uniformly available measured weight for the full sample (only a subsample had physical measures), so we relied on self-reports. We created a binary indicator for obesity (BMI ≥ 30 vs. <30). We also examined a three-category outcome: normal weight, overweight, obese, but for the primary analysis we focused on obese vs. not obese, given our interest in the health risk threshold and for ease of interpretation in regression models. At baseline 2014, we additionally calculated each person’s BMI and categorized them as obese or not, to use in descriptive transition analyses and as a covariate in some models.

#### Independent variable

Our focal independent variable was total household income. In HRS, income is reported as the total combined income of the respondent and (if applicable) spouse from all sources (earnings, pensions, Social Security, investments, etc.) in the previous calendar year. We used the measure of household income (in dollars) provided for each wave. Because income was highly skewed, we used two approaches in analysis: (1) a continuous measure after log-transformation, and (2) a categorical measure dividing respondents into income quartiles (four groups from lowest to highest income). The continuous log-income measure is convenient for regression modeling to detect linear trends [27], while the quartile categories are useful for descriptive comparisons and to allow potential non-linear effects [28] (for instance, if obesity risk drops sharply above a certain income threshold). In 2014, the median household income in our sample was approximately $50,000 (in 2018 dollars), with the lowest quartile roughly <$25,000 and the highest quartile >$90,000. We adjusted the data by capping extremely high incomes at the 99th percentile. This means that any income above this level was set equal to the value at the 99th percentile. We did this to prevent a few very large income values from skewing the results when analyzing income on a logarithmic scale.

#### Moderator

Race/ethnicity was our moderator. Each respondent’s race and ethnicity were self-identified. We created mutually exclusive categories: non-Hispanic White (reference group), non-Hispanic Black, and Hispanic (of any race). A small number of respondents identifying as other races were, as noted, excluded for this analysis due to insufficient sample for stable estimates. Race/ethnicity was treated as a time-invariant characteristic (assigned from baseline); we acknowledged that racial identity itself does not change, though the effects of race can accumulate over time. In our regression models, we included indicator variables for Black and for Hispanic, to compare each to the White group.

#### Control variable

To account for potential confounding in the relationship between income, race, and obesity, we included several demographic and health-related variables as covariates. Age was treated as a continuous variable, measured in years at each survey wave. This adjustment was necessary because weight patterns change with age; individuals in their 50s and 60s often continue gaining weight, while those in their 70s and older may begin to lose weight due to frailty or illness. Gender was included as a binary variable, with female coded as one and male as the reference category. This accounts for well-documented differences in obesity prevalence and the influence of socioeconomic status on health by gender. In older populations, women generally have higher obesity rates and different SES-health dynamics than men.

Nativity was considered by distinguishing between individuals born outside the United States and those born within. This was especially relevant given the ethnic diversity of the Hispanic population and the presence of foreign-born Black older adults, such as those from the Caribbean or Africa. Immigrant status is associated with different obesity trajectories, as foreign-born individuals often arrive with lower body weight but may experience weight gain over time due to acculturation and lifestyle changes [29]. Additionally, work status was measured as a time-varying indicator of whether the respondent was currently employed. This variable helps capture lifestyle differences, as those who are working may be more active or follow a structured routine, while those who are retired may either have more time for exercise or experience lower levels of activity. Since our income measure includes various sources such as wages, pensions, and Social Security, controlling for work status helps reduce bias from unmeasured health-related factors associated with employment.

Functional limitations were included to reflect physical health status. We used a binary measure indicating whether respondents had any difficulty with activities of daily living, such as bathing or dressing, or instrumental activities like walking several blocks or climbing stairs. These limitations were assessed at each wave and serve both as a potential consequence of obesity and a contributor to it. In 2014, about 30% of respondents reported at least one such limitation. Analyses using a count of limitations produced similar results. Finally, education was also included. Models used either years of education or grouped categories, including less than high school, high school graduate, some college, and college degree or higher. To avoid multicollinearity and keep the models parsimonious, we excluded education from the main results but confirmed that findings were robust when it was included. In the sample, 12% had less than a high school education, 59% had a high school diploma or some college, and 29% had a bachelor’s degree or more.

### Analytical plan

Our analysis proceeded in three main stages. First, we described baseline sample characteristics from 2014, stratified by race/ethnicity and income level. We reported obesity prevalence for White, Black, and Hispanic older adults across income quartiles and examined how obesity changed over time from 2014 to 2018. We calculated incidence (new cases) and remission (recovery) of obesity and explored how these transitions varied by race and income. To visualize trends, we plotted obesity prevalence by race and income over three survey waves, indicating whether disparities widened, narrowed, or remained stable over time.

The core of our analysis involved multivariable logistic regression for longitudinal (panel) data. We used mixed-effects models with random intercepts to account for repeated observations within individuals and to include both time-varying and time-invariant predictors, such as race. These models estimated how income is associated with the odds of obesity and how this relationship is moderated by race. Key interaction terms captured whether the protective effect of income differs for Black and Hispanic older adults compared to Whites. We reported odds ratios (ORs) with 95% confidence intervals (CIs), interpreting ORs below 1 as indicating a protective effect of income. Survey year indicators were added to adjust for secular trends in obesity between 2014 and 2018, and sensitivity checks using person fixed-effects and generalized estimating equations (GEE) confirmed the robustness of our findings.

To further explore how income and race interact, we conducted stratified models within each racial/ethnic group and by income levels. This allowed us to assess, for example, how income relates to obesity among Black seniors or how racial disparities in obesity differ between low- and high-income groups. We present the most relevant results in the main text, with additional models available upon request.

Finally, we estimated a lagged-dependent variable model to examine obesity transitions more directly. By predicting obesity status in 2018 while controlling for 2014 baseline status, we assessed whether income and race predicted becoming or ceasing to be obese. This helped reinforce the temporal ordering of our analysis. All models were implemented in Stata 16.

## Results

### Sample characteristics

Table 1 summarized the descriptive characteristics of the analytic sample in 2014, stratified by race, ethnicity, and household income level. The average respondent age was 67.4 years (standard deviation 10.2), and 55% were female. In terms of racial composition, 74% identified as non-Hispanic White, 16% as non-Hispanic Black, and 10% as Hispanic. Clear socioeconomic differences emerged: the median household income was $55,000 for White seniors, $35,000 for Black seniors, and $25,000 for Hispanic seniors. Educational attainment also varied significantly, with 20% of Black and 40% of Hispanic respondents lacking a high school diploma, compared to only 8% of Whites. These disparities reflect structural disadvantage experienced over the life course among minority older adults.

Obesity prevalence in 2014 was 33% across the full sample but varied by race. Black older adults had the highest rate at 42%, followed by Hispanic adults at 36%, and White adults at 30%. These differences represented substantial racial disparities. Black seniors were about 1.5 times as likely to be obese as White seniors, and Hispanic seniors about 1.2 times as likely. Despite being younger on average, White respondents had lower obesity rates. Gender-stratified analyses showed the racial gap was especially pronounced among women, with 48% of Black women obese compared to 32% of White women. Among men, the disparity was smaller, with 34% of Black men and 28% of White men classified as obese.

Household income was strongly linked to obesity. Those in the lowest income quartile, earning between $0 and $25,000 per year, had an obesity rate of 38%, compared to 27% in the highest income group, earning over $90,000 per year. This inverse gradient was strongest among White seniors: 35% of low-income White individuals were obese compared to 22% of those in the highest income group. For Black seniors, obesity prevalence remained high across income levels, with 45% in the lowest group and 38% in the highest, suggesting a weaker income gradient. Hispanic older adults showed some income-related differences, with 40% obesity in the lowest group and 28% in the highest, indicating a more moderate pattern.

Other covariates followed expected patterns. Older age was associated with lower obesity prevalence, with about 20% of individuals aged 80 and older classified as obese, compared to 37% among those in their 50s. Women had higher obesity rates than men, 36% versus 29%. Foreign-born respondents had lower obesity prevalence than U.S.-born individuals, 28% versus 34%, consistent with the healthy immigrant effect or cultural differences in diet and lifestyle. Those still working in 2014 had a slightly lower obesity rate of 30% compared to 35% among those not working or retired. Lastly, individuals with functional limitations had a substantially higher obesity rate of 45% compared to 27% among those without limitations, highlighting the relationship between physical impairments and obesity.

**Table 1:** Descriptive characteristics of older adults in HRS 2014, by race/ethnicity and income level

Characteristics (2014)	White	Black	Hispanic	Lowest Income Quartile	Highest Income Quartile
N (unweighted)	9,000	1,950	1,200	3,000	3,000
Age, mean (SD)	68.1 (10.3)	64.9 (9.4)	66.0 (9.8)	69.5 (11.2)	65.2 (9.1)
Female, %	53	60	58	57	51
Foreign-born, %	4	9	45	18	5
Working for pay, %	44	38	37	21	68
≥ 1 Functional limitation, %	28	36	32	50	15
**Household Income**, **median $**	$55,000	$35,000	$25,000	$12,000	$120,000
College degree or higher, %	31	15	10	5	60
**Obesity (BMI ≥ 30)**, **%**	30	42	36	38	27
– Men, %	28	34	30	35	25
– Women, %	32	48	40	40	29

Note: Data were from HRS 2014. The “Lowest Income Quartile” roughly corresponded to less than $25k per year, while the “Highest Income Quartile” corresponded to more than $90k per year. Percentages were weighted. Obesity was defined as BMI ≥ 30

### Longitudinal obesity transitions and trends (2014–2018)

Over the four-year follow-up, the overall prevalence of obesity in our sample increased slightly, from 33% in 2014 to 35% in 2018. This net change of + 2% points, though modest, is consistent with national trends of rising obesity among older cohorts. It is noteworthy that even in older age (when many people’s weights plateau or decline), the population-level obesity rate continued to edge upward, likely reflecting the higher BMI of incoming younger cohorts and the survival of those with obesity. To better understand this net change, we examined individual-level transitions in obesity status.

**Table 2:** Obesity transitions among older adults (2014–2018)

Group	Starting Proportion	Transition Outcome	Estimated Impact on Total Sample
Non-Obese in 2014	67%	8% became obese by 2018 (Incidence)	~ 5.4% of total transitioned to obese
Obese in 2014	33%	10% became non-obese by 2018 (Remission)	~ 3.3% of total transitioned to non-obese
Total Sample (2014–2018)	100%	Overall, obesity increased by 2.1% points	From 33–35% obesity prevalence

Among older adults who were not obese in 2014 (Table 2), about 8% transitioned into obesity by 2018. This meant that for every 100 older individuals who were of healthy weight or overweight at baseline, approximately eight became obese over the four-year period. The incidence of obesity was notably higher among younger seniors, particularly those in their 50s and early 60s, compared to those in their 80s, who were less likely to newly develop obesity and more likely to experience weight loss due to age-related factors. Equally, among those who were obese in 2014, accounting for about 33% of the sample, around 10% reduced their BMI to below 30 by 2018, transitioning out of the obese category. This remission likely reflected a mix of intentional weight loss and involuntary weight loss due to illness or mortality. Some individuals with severe obesity may not have survived through the follow-up period, which mathematically lowered the observed prevalence of obesity.

When these patterns were combined, the net increase in obesity prevalence became clear. The proportion who became obese, roughly 8% of 67% (about 5.4% of the total sample), was slightly larger than the proportion who ceased to be obese, about 10% of 33% (approximately 3.3% of the total). This led to a net gain of approximately 2.1% in the overall obesity rate, consistent with the observed rise from 33% in 2014 to around 35% in 2018.

### Multivariable regression results

Table 3 summarized the results of the mixed-effects logistic regression that predicted obesity based on income, race, and their interaction, while adjusting for all covariates. For ease of interpretation, the coefficients were presented as odds ratios (OR) with 95% confidence intervals. An OR above 1 indicated higher odds of obesity associated with that factor, while an OR below 1 indicated lower odds.

**Table 3:** Odds ratios from mixed-effects logistic regression of obesity on household income, race, and covariates (2014–2018)

Predictor	OR (95% CI)
**Household income (per $10**,**000)**	0.95 (0.92–0.99)**
**Black/African American (vs. White)**	1.50 (1.30–1.74)**
**Hispanic (vs. White)**	1.20 (1.02–1.41)*
**Income × Black interaction**	1.05 (1.01–1.09)*
**Income × Hispanic interaction**	1.03 (0.99–1.07)
Age (per year)	0.98 (0.97–0.99)**
Female (vs. Male)	1.11 (1.00–1.23)
Foreign-born (vs. U.S.-born)	0.85 (0.75–0.96)**
Currently working (vs. not)	0.90 (0.82–0.99)*
≥ 1 Functional limitation (vs. none)	1.29 (1.15–1.45)**
Wave 2016 (vs. 2014)	1.06 (0.99–1.13)
Wave 2018 (vs. 2014)	1.10 (1.02–1.18)*
**N (individuals)**	12,118
**Observations (person-waves)**	33,856
**Log Likelihood**	–18,234

*p* < 0.05, *p < 0.01. OR = Odds Ratio. Income is measured in 2018 dollars, inflation-adjusted; OR is per additional $10,000/year in household income (income was mean-centered, so main effects of Black/Hispanic reflect comparison at average income). Random intercept for person included. Model also adjusted for education in a sensitivity run (not shown) with minimal changes in coefficients

Household income showed a significant inverse association with obesity odds. For every $10,000 increase in annual income, the odds of being obese decreased by approximately 5% (OR = 0.95, 95% CI: 0.92–0.99, *p* < 0.01), holding all other factors constant. For instance, a $50,000 difference in income (roughly spanning the bottom to the top quartile) translated to about 23% lower odds of obesity (OR ≈ 0.77). This supported the expected socioeconomic gradient in obesity, although this average effect primarily reflected the White reference group. Racial disparities in obesity persisted after adjusting for income and covariates. Black older adults had 1.50 times the odds of obesity compared to Whites (OR = 1.50, CI: 1.30–1.74, *p* < 0.01), and Hispanic older adults had 1.20 times the odds (OR = 1.20, CI: 1.02–1.41, *p* < 0.05). These figures represented disparities at the mean income level and indicated that Black and Hispanic seniors remained at elevated obesity risk even after accounting for other factors.

Interactions between income and race revealed that the protective effect of income varied by racial group. Among Black older adults, the income-obesity association was significantly weaker (interaction OR = 1.05, CI: 1.01–1.09, *p* < 0.05). For Black seniors, the overall effect of income on obesity was nearly null (adjusted OR ≈ 1.00), meaning that additional income did not significantly reduce obesity risk. For instance, a $50,000 increase in income resulted in only a marginal change in obesity odds for Blacks (OR ≈ 0.98), whereas the same income increase among Whites resulted in a 23% reduction. For Hispanic seniors, the interaction term was positive but not statistically significant (OR = 1.03, CI: 0.99–1.07, *p* = 0.10), suggesting a modest attenuation of the income effect. When model predictions were applied, at a low income level ($20,000), predicted obesity prevalence was about 35% for Whites, 45% for Blacks, and 38% for Hispanics. At a high income level ($100,000), obesity risk fell to 25% for Whites, 42% for Blacks, and 34% for Hispanics. Thus, income gradients were steep among Whites, moderate for Hispanics, and nearly flat for Blacks. Stratified models further confirmed this pattern: among high-income individuals, Blacks had twice the odds of obesity compared to Whites, while the disparity was smaller among low-income groups. This supported the notion of “emergent inequality” at higher SES, where racial health gaps widen as Whites accrue greater health returns from income.

Covariate effects aligned with expectations. Age was associated with lower obesity odds (OR = 0.98 per year, CI: 0.97–0.99, *p* < 0.01), suggesting a 20% decrease in obesity likelihood over a decade. Gender showed a modest effect: women had slightly higher odds of obesity than men (OR = 1.11, CI: 1.00–1.23, *p* = 0.05), consistent with patterns observed in prior studies. Foreign-born seniors had lower obesity risk (OR = 0.85, CI: 0.75–0.96, *p* < 0.01), which supported the healthy immigrant hypothesis. Those currently employed had somewhat lower odds of obesity (OR = 0.90, CI: 0.82–0.99, *p* < 0.05), possibly reflecting better functional health or lifestyle structure. Individuals with at least one functional limitation had significantly higher obesity odds (OR = 1.29, CI: 1.15–1.45, *p* < 0.01), which underscored the bi-directional relationship between obesity and mobility impairments.

Time trends revealed that the odds of obesity in 2018 were about 10% higher than in 2014 (OR = 1.10, CI: 1.02–1.18, *p* < 0.05), even after adjusting for other variables, consistent with rising obesity prevalence nationally. The 2016 wave did not differ significantly from 2014, indicating that most of the increase occurred between 2016 and 2018.

To test the robustness of our findings, we conducted several checks. First, using income quartiles instead of a continuous measure showed similar results: White seniors in the highest quartile had 18% lower obesity odds than those in the lowest (OR = 0.82, *p* < 0.01), while the effect for Black seniors was weaker and not statistically significant (OR ≈ 0.95). Second, we addressed potential attrition bias by applying HRS-provided weights and comparing results to cross-sectional models from 2014, which produced consistent patterns. Third, a fixed effects logistic model, which focused on within person changes, found no significant income effect, likely because income did not vary much among older adults. However, the model did show weight gain among those who transitioned from work to retirement, suggesting that the observed income–obesity relationship primarily reflected long-term socioeconomic differences rather than short-term income changes in later life.

**Fig. 1:**
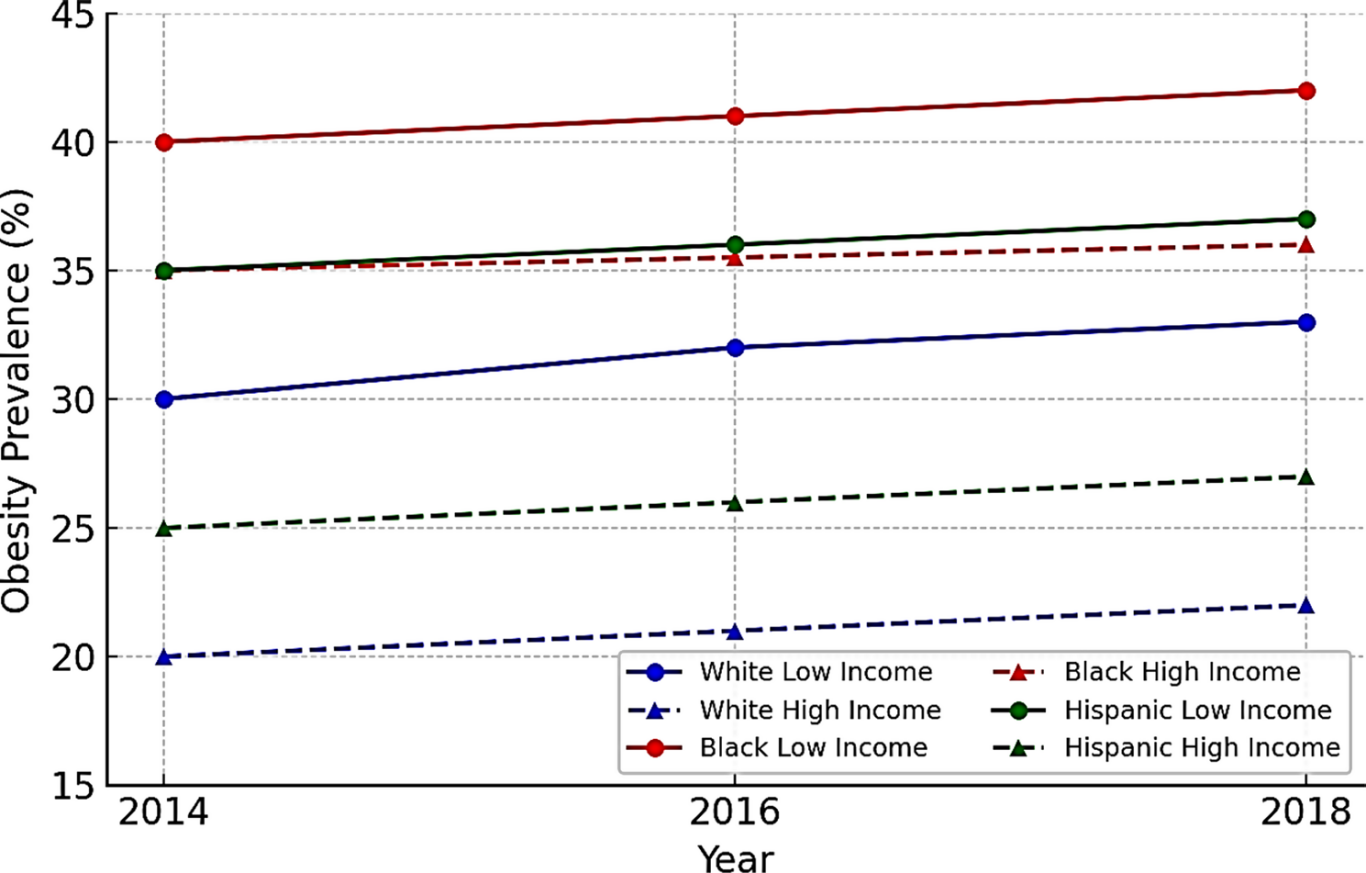
Obesity prevalence 2014–2018 by race and income group

Figure 1 showed that Black older adults consistently had the highest obesity prevalence across all income levels. Between 2014 and 2018, obesity among low-income Black seniors rose slightly from 40 to 42%, and among high-income Black seniors from 35 to 36%, reflecting a small income gap. In contrast, White older adults showed a clear income gradient: obesity was 30–33% among low-income Whites and only 20–22% among high-income Whites, a 10-point difference. Hispanic seniors were in between, with a gap of around 10% points as well rising from 35 to 37% in the low-income group and 25–27% in the high-income group. Racial disparities remained evident over time. In 2018, high-income Black seniors still had a higher obesity rate (36%) than high-income Whites (22%), and low-income Blacks (42%) had higher rates than both Hispanics (37%) and Whites (33%). While obesity rose slightly across all groups, the disparities persisted. The income-obesity gap was steepest for Whites, moderate for Hispanics, and minimal for Blacks. These patterns laid the groundwork for our multivariable analysis to examine whether income effects on obesity differed significantly by race.

## Discussion

This study aimed to explore the relationship between household income and obesity among older adults and to understand how this relationship varies across racial and ethnic groups. We found that higher income does not consistently lead to better obesity outcomes for all populations. Notably, while higher income was strongly associated with lower obesity risk among White older adults, it had little effect on Black older adults. Hispanic seniors fell in between, exhibiting a modest benefit.

Our results confirmed prior research showing that older adults with greater financial resources tend to have lower obesity rates [30, 31]. This finding supports Grossman’s health capital theory, which posits that individuals who can afford nutritious food, time for physical activity, and quality healthcare are more likely to maintain a healthy weight [32]. It also aligns with the social determinants of health framework, emphasizing how income facilitates access to health-promoting environments and buffers against stress-related weight gain [33]. Importantly, our study demonstrates that this socioeconomic gradient persists into older adulthood. Seniors with consistent income from pensions, investments, or continued employment tended to have healthier weight status. However, the magnitude of the income effect was moderate, and it is evident that financial resources alone cannot fully explain obesity in late life. Biological aging, medication side effects, and earlier life exposures also play a critical role.

One of the most salient findings was the way race shaped the income obesity relationship. For White seniors, higher income was clearly associated with lower obesity prevalence, 22% among high-income Whites compared to 33% among their low-income counterparts in 2018. For Black seniors, however, income level made little difference. Their obesity rates ranged from 36 to 42% regardless of income. This pattern aligns with the theory of “diminishing returns,” which suggests that socioeconomic gains do not translate equally across racial groups [33]. Despite comparable education and income, Black Americans often experience worse health outcomes than their White peers [34].

Several factors help explain why income does not offer equal health benefits across racial groups. Older Black adults often face lifelong exposure to structural discrimination and chronic stress, a process known as weathering, which can lead to biological changes that increase obesity risk [35]. Even with higher income, these cumulative effects may persist. Additionally, due to residential segregation and historical housing discrimination, many high-income Black seniors live in under-resourced neighborhoods with limited access to healthy food and safe spaces for physical activity [36]. In contrast, high-income White seniors are more likely to live in areas with health-promoting infrastructure, making it easier for them to convert economic advantage into better health [18].

Cultural perceptions of body size and health may further shape these outcomes. Research has found that Black and Hispanic adults, especially women, are more likely to view larger body sizes as acceptable [37]. In some cultural contexts, being “fuller” is not stigmatized and may be perceived as a sign of well-being [38]. As a result, individuals whose social networks do not emphasize weight loss may be less inclined to pursue it, even if they have the means to do so. Dietary traditions also contribute; culturally valued foods such as soul food or calorie-dense Hispanic dishes may remain prevalent across income levels [39]. In contrast, higher SES White adults often adopt distinct eating patterns, including organic or Mediterranean-style diets, which may contribute to lower obesity rates [40].

Hispanic older adults had slightly higher adjusted odds of obesity than Whites, although their income obesity association was weaker. The so-called “Hispanic paradox,” whereby Hispanics have health outcomes comparable to or better than Whites despite lower income, was not clearly evident in obesity outcomes [41]. Instead, nativity status was a key differentiator: foreign-born Hispanics tended to have lower obesity prevalence than their U.S.-born counterparts [42]. This suggests that cultural acculturation may erode the health advantages of higher income among Hispanics by promoting adoption of more sedentary lifestyles and processed diets common in the U.S.

Healthcare access and utilization may also play a role. Although higher income typically facilitates access to medical care, high-income Black individuals may be less inclined to engage with weight management services if they have experienced discrimination or distrust within the healthcare system. Research indicates that Black patients are less likely to receive obesity-related counseling, even when clinically appropriate [43]. Concerns about cultural sensitivity may also lead healthcare providers to avoid addressing weight, resulting in missed opportunities for intervention.

## Limitations

While this study provides important findings, several limitations should be noted. The study did not include direct measures of key behavioral and environmental factors such as diet quality, physical activity, or neighborhood food environments. These factors are likely to vary systematically by income and race, and their absence limits our ability to fully explain the observed associations. While we referenced prior literature on structural barriers, cultural norms, and environmental conditions to contextualize our results, our analysis could not directly test these pathways. Additionally, both the outcome and some predictor variables may be prone to measurement limitations. Obesity was defined using BMI derived from self-reported height and weight, which can be subject to recall or reporting errors, especially in older adults who may underestimate weight or overestimate height due to age-related changes. Although BMI is widely used, it does not distinguish between fat and muscle mass, which may lead to misclassification, particularly in aging populations with muscle loss. Income was measured as current household income, which does not fully capture lifetime socioeconomic position or accumulated wealth. Some older adults may have low income but substantial assets, while others may experience financial hardship despite having had higher earnings earlier in life.

The relatively short follow-up period of four years may have limited our ability to detect long-term trends or more gradual effects of socioeconomic change. Behavioral shifts and weight outcomes often unfold over longer periods. Furthermore, attrition due to mortality or non-response between survey waves may have biased the results, especially if those who dropped out were disproportionately obese or of lower socioeconomic status. Although we applied longitudinal weights to adjust for known attrition patterns, unknown biases could persist.

Our findings may also have limited generalizability. The analysis focused on U.S. adults aged 50 and older who identified as non-Hispanic White, non-Hispanic Black, or Hispanic. Within-group heterogeneity could not be fully captured; for example, Hispanic respondents included individuals from diverse national backgrounds, and Black respondents included both African Americans and immigrants. Additionally, the study did not examine other racial or ethnic groups such as Asian Americans or Native Americans, who may experience different socioeconomic and health dynamics. Finally, survival bias is a potential concern. Individuals with the most severe obesity or associated illnesses may not have lived to be included in the sample, particularly among low-income and minority populations. This could lead to underestimation of obesity disparities in later life.

## Conclusion and policy implications

This study revealed that higher income is associated with lower obesity risk among older adults, but this benefit is uneven across racial and ethnic groups. While White seniors experienced a clear protective effect, Black seniors showed no significant reduction in obesity with increased income, and Hispanic seniors saw only modest gains. These patterns reflect broader structural inequalities and point to the limitations of income alone in addressing health disparities in later life. Effective policy responses must begin with culturally tailored interventions. Programs delivered through trusted community venues, such as churches or senior centers, should incorporate familiar foods and social support to better engage Black and Hispanic older adults. Efforts that align with cultural norms, such as Spanish-language outreach or culturally meaningful activities like dance classes, can improve participation and outcomes.

At the same time, improving neighborhood environments is essential. Many older adults in minority communities lack access to healthy food, safe places to walk, or recreational spaces. Policies should expand access to grocery stores, parks, and infrastructure that supports older adults, while supporting programs like SNAP to make healthy options more affordable and accessible. Additionally, healthcare systems must also play a proactive role. Providers should deliver weight management counseling that is sensitive to cultural differences and ensure that minority seniors are included in preventive programs such as the Medicare Diabetes Prevention Program. Building trust through provider diversity and community partnerships is key to improving engagement.

Finally, addressing disparities requires long-term investment in policies that promote equity and social mobility. Interventions earlier in life that reduce poverty and expand educational opportunities can yield health benefits in later years. In the short term, expanding income support, nutrition assistance, and healthcare access for low-income seniors can help reduce the burden of obesity. A coordinated and comprehensive approach involving public health, healthcare, and community planning is essential to ensure that all older adults, regardless of race or income, have the opportunity to age with good health and dignity.

## Data Availability

The data for this study were obtained from the publicly available Health and Retirement Study (HRS) dataset. All analyses were performed using publicly available data, and no additional permissions were required for their use. Researchers interested in replicating or extending the findings are encouraged to access the data through the HRS repository.

## Abbreviations

BMI: Body Mass Index
HRS: Health and Retirement Study
SES: Socioeconomic Status
